# Collodion Controversy

**Published:** 1848-07

**Authors:** 


*Collodion Controversy.*—The controversy between two medical students of Boston, as to the merit of priority in having accidentally dropped a little ethereal solution of gun cotton on the finger, and discovered it to be an adhesive medium, bids fair to rival the gas war. Our friend the Editor of the Boston Journal, has placed his veto on any farther discussion of the subject in his pages. If we might presume to advise, it would be that the young gentlemen direct their enquiries to ascertain the extent to which the article is really useful. It may be that victory is not worth the powder. The discussion, if intended as a satire upon that which preceded it, would be excellent. Ether applied upon the surface, as well as respired, seems to have a peculiar effect in confusing the memory and blunting the judgment.

